# Fibrotic microRNAs in the suppression of HSC activation and ECM deposition to facilitate the regression of hepatic fibrosis in zebrafish

**DOI:** 10.1007/s00109-026-02672-y

**Published:** 2026-04-30

**Authors:** Yu-Heng Lai, Mu-Kuang He, Chung-Tsui Huang, Han-Yu Tseng, Tzu-Ching Lin, Ting-Yu Yang, Semon Wu, Guor Mour Her

**Affiliations:** 1 Department of Chemistry, Chinese Culture University, 55, Hwa-Kang Rd. Yang-Ming-Shan, Taipei, 111 Taiwan; 2 School of Medicine, College of Medicine, Taipei Medical University, No.250, Wuxing St., Xinyi Dist., Taipei, 110 Taiwan; 3 Department of Internal Medicine, Division of Gastroenterology and Hepatology, Far Eastern Memorial Hospital, No. 21, Section 2, Nanya S. Road, Banqiao District, New Taipei, 220 Taiwan; 4 Institute of Biopharmaceutical Sciences, National Yang Ming Chiao Tung University, No.155, Sec.2, Linong Street, Taipei, 112 Taiwan; 5 Department of Life Science, Chinese Culture University, 55, Hwa-Kang Rd., Yang-Ming-Shan, Taipei, 111 Taiwan

**Keywords:** Liver fibrosis, TGFβ-1, MiRNA, Zebrafish

## Abstract

**Abstract:**

During liver injury, early pathological changes may go unnoticed, delaying the diagnosis until the disease has advanced to stages such as fibrosis, cirrhosis, or hepatocellular carcinoma. Hepatic fibrosis is a sign of long-term liver injury that leads toward permanent liver damage. It involves the buildup of too much extracellular matrix (ECM) and is activated by hepatic stellate cells (HSCs). The transforming growth factor-β1 (TGF-β1)/SMAD pathway is central to this process, which shows great potential for therapeutic strategies. Therefore, we investigated the therapeutic potential of microRNA (miRNA)-mediated regulation of fibrogenesis by using zebrafish models. A group of fibrosis-related miRNAs was identified and used to generate transgenic zebrafish lines. Fibrosis was induced via doxycycline (DOX)-controlled overexpression of TGF-β1a, and the antifibrotic efficacy of miRNAs was evaluated. Quantitative real-time PCR showed a marked downregulation of the target miRNAs, accompanied by decreased expression of fibrotic markers. Next, we confirmed that TGF-β1a, GREM1, and α-SMA protein levels were decreasing. Histological staining revealed improved liver structure and diminished collagen deposition. These findings indicate that through manipulating the miRNA expressions, we can effectively disrupt TGF-β-induced fibrogenesis, inactivate HSC, inhibit ECM remodeling, and attenuate liver fibrosis, and highlight the potential of miRNA-based therapeutic strategies for early intervention in liver disease.

**Key messages:**

- Establishment of an *in vivo* model for developing an inducible miRNA expression system in zebrafish.
- Predicting and evaluating targeted miRNAs that attenuate TGF-β-induced hepatic fibrosis.
- Elucidate the potential mechanism for fibrosis that may be a strategy for future therapies.

**Graphical Abstract:**

MicroRNA-mediated modulation attenuates hepatic fibrosis by coordinately suppressing the TGF-β/SMAD signaling pathway, hepatic stellate cell activation, and extracellular matrix deposition. Protein-level validation confirms reduced phosphorylation of Smad2 and decreased expression of key fibrotic markers, including α-SMA, collagen I, and fibronectin. These findings highlight a multi-target regulatory mechanism through which miRNAs exert antifibrotic effects at the systems level.

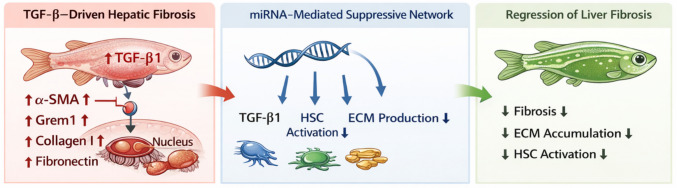

**Supplementary Information:**

The online version contains supplementary material available at 10.1007/s00109-026-02672-y.

## Introduction

Hepatic fibrosis is one of the progressive processes that forms chronic liver disease. It develops through a series of harmful events mainly caused by oxidative stress and long-term inflammation. In the beginning, liver damage leads to the release of reactive oxygen species (ROS) from hepatocytes. This process activates dormant hepatic stellate cells (HSCs) and draws in tissue macrophages via the CCL2-CCR2 pathway [1]. In the second stage, activated macrophages and HSCs keep releasing pro-inflammatory cytokines. These include transforming growth factor-β (TGF-β), interleukin-6 (IL-6), interleukin-1β (IL-1β), and tumor necrosis factor-α (TNF-α) [2]. These signaling molecules increase the interaction between hepatocytes and urge HSCs to turn into myofibroblasts. Platelet-derived growth factor (PDGF) also stimulates myofibroblasts to grow. This leads to the overproduction and build-up of extracellular matrix (ECM) components, such as type I collagen and α-smooth muscle actin (α-SMA) [3–5]. During the final stage, as myofibroblasts accumulate, the composition of ECM remodels from type IV and VI collagen, becoming a matrix dominated by type I and III collagen, which is more resistant to protease degradation [6]. The collagen fibers are cross-linked and activated by lysyl oxidase and form dense fibrotic tissue that replaces normal hepatic structure [7, 8]. ECM accumulation and replacement continuously disrupt the structure and function of the liver, which leads to fibrosis and progresses to cirrhosis [9, 10].

TGF-β is a pivot in the progression of liver fibrosis that shows therapeutic potential [11]. It is a cytokine that involves facilitating cell proliferation, differentiation, ECM composition, and collagen formation [12]. TGF-β is first translated as a precursor complex. The mature, active 25-kDa homodimer is then cleaved out of the pro-peptide region, called a latency-associated peptide (LAP), but is non-covalently attached to the small latent complex (SLC). In the endoplasmic reticulum, SLC associates with latent TGF-β binding protein (LTBP) to create the large latent complex (LLC), which is subsequently integrated into the ECM and keeps TGF-β inactive [13]. Various mechanisms tightly control the activation of TGF-β. For example, integrins can bind LAP and release active TGF-β [14]. Moreover, proteolytic enzymes such as plasmin, MMP-2 [15], MMP-9 [16], BMP-1 [17], and plasma kallikrein [18] are able to cleave LAP to release active TGF-β and result in the activation of LTBP and ECM maturation [12]. When the mature TGF-β ligand docks on type I and type II TGF-β receptors on the cell surface to become a heteromeric complex, the TGF-β-mediated fibrosis signaling cascade is triggered. Upon ligand binding, the phosphorylation of type II receptor and the type I receptor activation phosphorylates receptor-regulated SMAD proteins (R-SMADs), mainly SMAD2/3 or SMAD1/5/8. Activated R-SMADs form a complex with SMAD4 and translocate into the nucleus to trigger the downstream pathway [19, 20]. I-SMADs, SMAD6 and SMAD7, work as inhibitory SMADs, showing the competitive activity with R-SMADs to bind to the type I receptor, which reduces downstream signaling [21, 22]. Additionally, SMAD7 directly inhibits the transcriptional activity of SMAD complexes in the nucleus, which provides negative feedback in the TGF-β pathway.

On the other hand, during fibrosis, matrix metalloproteinases (MMPs) are a group of zinc-dependent proteases that are crucial in ECM decomposition and reconstruction [23, 24]. During collagenesis regulation, specific MMPs, such as MMP-2, MMP-9, MMP-13, and MMP-14, degrade type I and III collagens and activate each other in a regulatory network [25]. Furthermore, tissue inhibitors of metalloproteinases (TIMPs), especially TIMP-2, block MMP-14 and activate other MMPs [26, 27] to inhibit the proteolytic interaction. Through various cytokine signaling, protease activity, and ECM interactions, hepatic fibrosis proceeds with the continuous activation of HSCs and excessive ECM deposition.

MicroRNAs (miRNAs) have been considered potential therapeutic agents for liver fibrosis [26]. MiRNAs can significantly attenuate fibrotic processes by regulating critical pathways in HSC activation. For example, miR-214 has been identified to promote liver fibrosis by activating HSCs through the suppression of the negative regulator Sufu, which suggests that inhibition of miR-214 restores the fibrosis progression [28]. Additionally, miR-223 expression demonstrates antifibrotic effects, which are referred to as a potential therapeutic candidate [29]. Recent evidence showed that miR-155 is a critical regulator that may attenuate liver fibrosis [30]. Several pathways for regulating miRNA activity have been investigated. For example, the miRNA sponge technique is to design a loss of function of miRNAs in cells [31]. When miRNA sponges are applied, which bind to the target miRNA in the seeding region specifically, they can effectively shut down the whole transcripts of an entire family of related miRNAs [32]. These studies showed the clinical potential of miRNA-associated therapies in treating liver fibrosis and preventing progression to liver cirrhosis [33, 34]. Thus, miRNAs provide a means to directly modulate the TGF-β/SMAD pathway, thereby regulating HSC activation and liver fibrosis inhibition. Furthermore, the balance between disrupting and remodeling of fibrotic ECM through the MMP pathway may also ultimately achieve a regression of fibrosis.

In summary, our study highlights the suitability of zebrafish as an in vivo model for developing an inducible miRNA expression system. The results demonstrate that targeted miRNA expression significantly attenuates TGF-β-induced hepatic fibrosis by suppressing HSC activation and ECM deposition, ultimately facilitating fibrosis regression. These findings underscore the therapeutic promise of modulating the TGF-β/Smad signaling axis via miRNA-based interventions, presenting a compelling strategy for future antifibrotic therapies.

## Materials and methods

### Zebrafish husbandry and doxycycline treatment

Zebrafish were cultured in a recirculating aquatic system with a light–dark cycle of 14/10 h, with the water temperature at 28 °C. The acidity range of the water was from pH 6.5 to 6.8. The zebrafish were fed three times a day with regular fish flakes. At 10-day-post-fertilization (dpf) and 1-month-post-fertilization (mpf), zebrafish were immersed to 40 µg/ml of doxycycline (DOX).

### Functional analysis of the selective fibrotic miRNAs

We started with literature searching for functional analysis of miRNAs on their related functions (http://www.ncbi.nlm.nih.gov/pubmed). In addition, we used two target gene prediction databases: TargetScan (http://www.targetscan.org) and miRTarbase (http://mirtarbase.mbc.nctu.edu.tw) to identify target genes that overlapped with these two databases for further analysis. The two databases were used to mine the conservation similarity of zebrafish and human miRNAs, and finally filtered into miR-454b, miR-190a, miR-96, and miR-196a as OE^TSP^ constructs. At the same time, miR-21, miR-25, miR-92a, miR-155, and miR-183 were designed as SP^TSP^ constructs (Table 1). MiR-29b, miR-153a-3p, and miR-204-5p were chosen for miRNA design as OE^ECM^ constructs. MiR-34a, miR-183-5p, miR-150, miR-193a-3p, and miR-125b-5p were selected to be designed as SP^ECM^ constructs (Table 2, Fig. S1).

**Table 1:** Systematic predictive results of fibrotic miRNA-OE^TSP^ and miR-SP^TSP^. Fibrotic miRNAs were predicted through databases comparison of human and zebrafish

Human	Zebrafish	miRNA
Overexpression (OE^TSP^)		
*tgf-β1*	*tgf-β1a*	miR-454b
*smad3*	*smad3a*	miR-190a
	*smad3b*	miR-96
*smad4*	*smad4a*	miR-196a
Sponge (SP^TSP^)		
*smad7*	*smad7*	miR-25/92a
*tgif1*	*tgif1*	miR-25/92a
*ski*	*skia*	miR-155
	*skib*	miR-21
*snon*	*snona*	miR-155
	*snonb*	miR-183

*OE* overexpression, *SP* sponge, *TSP* TGF-β/SMAD pathway

**Table 2:** Systematic predictive results of fibrotic miRNA-OE^ECM^ and miR-SP^ECM^. Fibrotic miRNAs were predicted through database comparison of human and zebrafish

Human	Zebrafish	miRNA
Overexpression (OE^ECM^)		
*loxl2*	*loxl2b*	miR-29b
*timp2*	*timp2a*	miR-153a-3p
		miR-204-5p
Sponge (SP^ECM^)		
*mmp-2*	*mmp2*	miR-34a
*mmp-9*	*mmp9*	miR-183-5p
*mmp-13*	*mmp13b*	miR-150
*mmp-14*	*mmp14a*	miR-193a-3p
	@	miR-125b-5p

*OE *overexpression, *SP* sponge, *ECM* extracellular matrix-associated pathway

### Plasmid construction and transgenic zebrafish line generation

Sequence of four recombinases was synthesized (Genomics BioSci & Tech., Taipei, Taiwan) to generate different kinds of recombinase lines and cloned into the plasmid with liver fatty acid binding protein (L-FABP) promoter and Tet-On system [35–38]. Four recombinase lines are abbreviated as LF-ON3-Cre-eR (*Tg(fabp10a:Tet*^*on*^*-Tre3-Cre*) crossed with *Tg*(*Cryb:zmCherry*) carries Cre recombinase and exhibits red eye fluorescence); LF-ON3-zDre-eG (*Tg*(*fabp10a:Tet*^*on*^*-Tre3-Dre*) crossed with *Tg*(*Cryb:GFP*) carries Dre recombinase and exhibits green eye fluorescence); LF-ON3-FLP-hR (*Tg*(*fabp10a:Tet*^*on*^*-Tre3-Flp*) crossed with *Tg*(*Myl7:zmCherry*) carries Flp recombinase and exhibits red heart fluorescence), and LF-ON3-Vika-hG (*Tg*(*fabp10a:Tet*^*on*^*-Tre3-Vika*) crossed with *Tg*(*Myl7:GFP*) carries Vika recombinase and exhibits green heart fluorescence). The zebrafish with zCre transgene displayed red fluorescence in their eyes, while those with zDre transgene exhibited green fluorescence. Zebrafish carrying the zFLP transgene displayed red fluorescence specifically in their hearts, whereas those with the Vika transgene exhibited green fluorescence.

To generate miRNA regulation lines, LRFV sequence including cutting sites of the four recombinases (loxP, rox, FRT, and vox) was synthesized (Genomics BioSci & Tech., Taipei, Taiwan). The LRFV sequences were cloned into the plasmid along with the L-FABP promoter and two genes of fluorescence proteins (CFP and mCherry), respectively. Liver-specific miRNA regulation lines were abbreviated as OE^TSP^ (*Tg*(*fabp10a-LRFV-R-miRNA-OE*^*TSP*^); SP^TSP^ (*Tg*(*fabp10a-LRFV-R-miRNA-SP*^*TSP*^); OE^ECM^ (*Tg*(*fabp10a-LRFV-miRNA-OE*^*ECM*^), and SP^ECM^ (*Tg*(*fabp10a-LRFV-miRNA-SP*^*ECM*^) lines.

### Histological staining

Each zebrafish was subsequently fixed in 10% paraformaldehyde/PBS and embedded in paraffin for staining with Masson’s trichrome staining (Merck, MI, USA). All specimens were observed microscopically (Leica, Tokyo, Japan) at ×200 magnification.

### Quantitative real-time reverse transcription polymerase chain reaction (qRT-PCR)

Total RNA of tissue was harvested by using Trizol (Merck, MI, USA), and reverse transcription was performed using SuperScript II transcriptase (Thermo Fisher Scientific, MA, USA). The amount of liver tissue and RNA for reverse transcription was followed by kit protocols, in which we extracted RNA from half of the liver tissue and applied 2 μg of RNA to perform reverse transcription. Quantitative real-time PCR (qRT-PCR) was performed using the StepOne™ System (Thermo Fisher Scientific, MA, USA). The temperature setting was followed by the product manual, except that the heat annealing temperature was set at 58 °C for 35 cycles. All qRT-PCR data were calculated using the 2^-ΔΔCt method, normalized to U6 or *gapdh*, and expressed as fold change relative to the control (NC) group, which was set to 1. All primer sequences used in this study are listed in Table S1.

### Western blotting

An equal amount of proteins was loaded and separated by 10% SDS–polyacrylamide gel and transferred to polyvinylidene difluoride (PVDF) membranes. The membranes were incubated with the primary and secondary antibodies for 1 h. The proteins were detected using the enhanced ECL chemiluminescence Western Blotting Detection System (ChemiDoc XRS, Bio-Rad Laboratories, CA, USA). Signal strengths were quantified using a densitometric program (Bio-Rad Laboratories, CA, USA).

### Statistical analysis

All values are expressed as means ± standard deviation (SD) with at least three independent experiments. GraphPad Prism 9.0 software (GraphPad, San Diego, California, USA) with analysis of variance (ANOVA) followed by Bonferroni post hoc test was used to compare differences among groups of samples. Asterisks were used to indicate that a value was significantly different from the control.

## Results

### Selection of fibrotic miRNA to design the miRNA regulation construct

We browsed the database of miRNA targeting sequences between human and zebrafish to design miRNA overexpression (OE) and miRNA sponge (SP) inhibitory constructs. To evaluate the TGF-β pathway in the liver fibrosis network, zebrafish genes, *tgf-β1a*, *smad3a*, *smad3b*, *smad4a*, *smad7*, *tgif1*, *skia*, *skib*, *snona*, and *snonb* were first investigated through TargetScanFish. In addition, miRbase.org was used to mine the conservation similarity of zebrafish and human miRNAs, and finally filtered into miR-454b, miR-190a, miR-96, and miR-196a as OE^TSP^ constructs, while miR-21, miR-25, miR-92a, miR-155, and miR-183 were designed as SP^TSP^ constructs (Table 1).

On the other hand, we dissected the MMP-associated pathway in fibrosis and revealed that *loxl2b* and *timp2a* exhibited higher similarity between zebrafish and human. MiR-29b, miR-153a-3p, and miR-204-5p were chosen for miRNA design as OE^ECM^ constructs. Zebrafish *mmp2*, *mmp9*, *mmp13b*, and *mmp14a* also displayed greater similarity to human. Therefore, miR-34a, miR-183-5p, miR-150, miR-193a-3p, and miR-125b-5p were selected to be designed as SP^ECM^ constructs (Table 2, Fig. S1).

### Generation of tissue-specific and inducible miRNA transgenic zebrafish

**Fig. 1:**
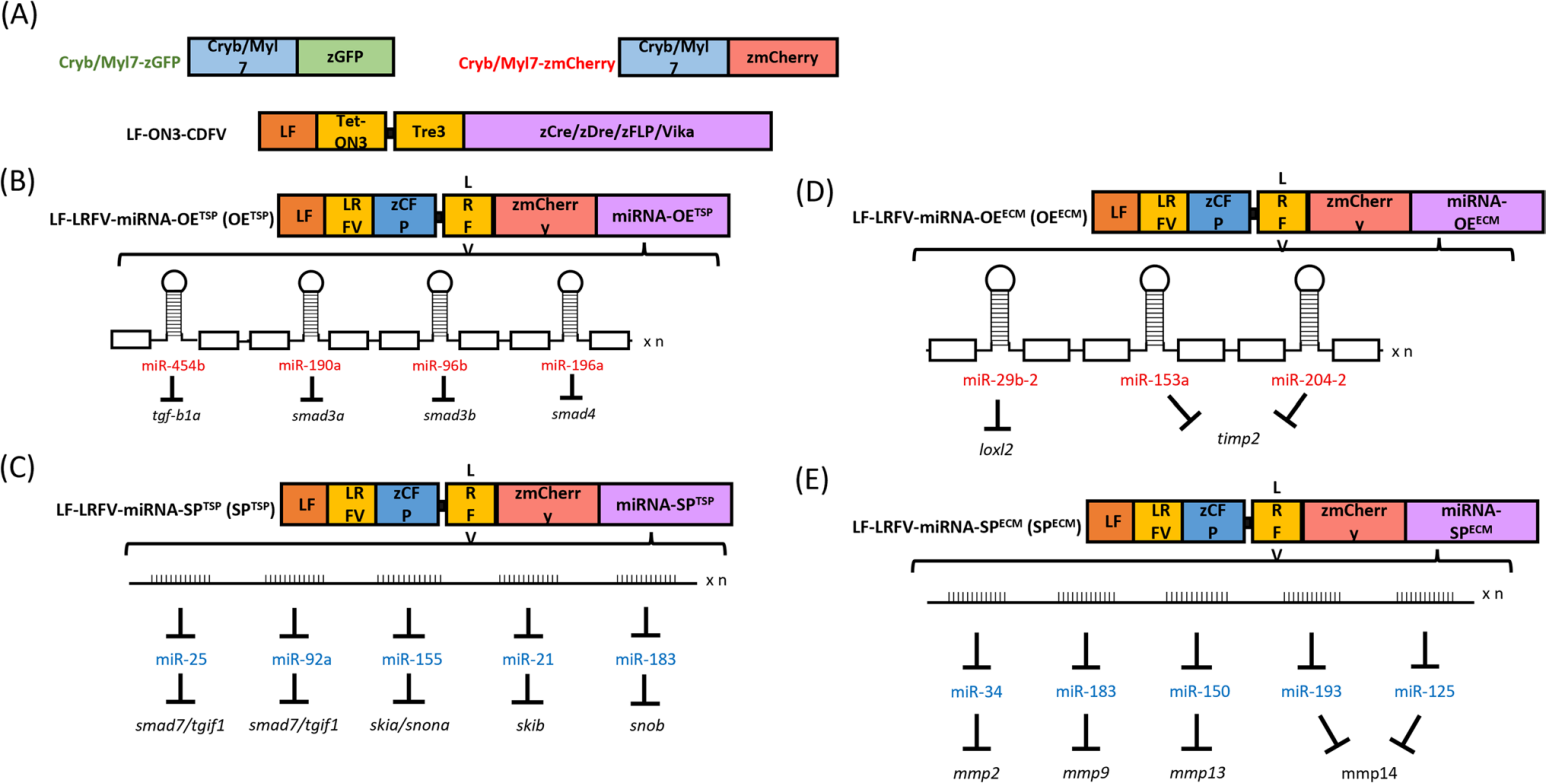
Scheme of establishment of miRNAs expressing tissue-specific zebrafish lines. **A** The recombinase construct consists of the eye-specific promoter (Cryb), the heart-specific promoter (Myl7), and four types of recombinases (zCre/zDre/zFLP/Vika). **B** The LF-LRFV-miRNA-OE^TSP^ construct contains a liver-specific promoter (liver fatty acid binding protein), CFP, zmCherry, and miRNA-OE^TSP^ with the corresponding targeted mRNAs. **C** The LF-LRFV-miRNA-SP^TSP^ construct contains two copies of sponge sequence. **D** The LF-LRFV-miRNA-OE^ECM^ construct. **E** The LF-LRFV-miRNA-SP^ECM^ construct. LF, liver fatty acid binding protein promoter; LRFV, recombinase cutting sites loxP, rox. z-, sequences that are optimized for translation in zebrafish; CFP, cyan fluorescent protein; mCherry, red fluorescent protein; ECM, extracellular matrix

**Fig. 2:**
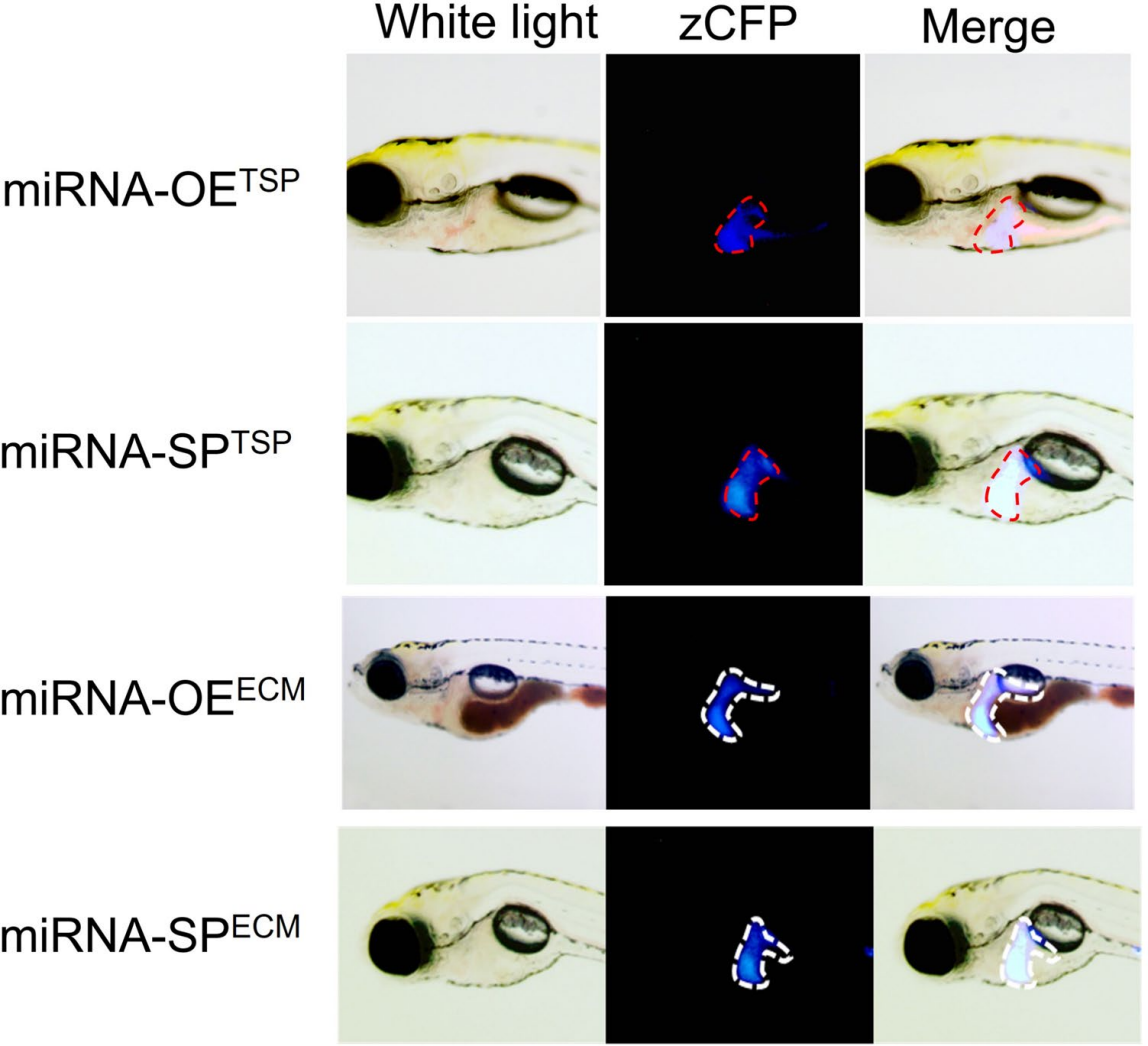
Expression of miRNA- OE^TSP^/SP^TSP^ or miRNA- OE^ECM^/SP^ECM^ in zebrafish larvae. At 7 dpf, larvae were observed under a fluorescence microscope. In overlapped images, a blue fluorescent signal from CFP was observed in the liver region. OE, overexpression; SP, sponge; TSP, TGF-β/SMAD pathway; ECM, extracellular matrix-associated pathway

**Fig. 3:**
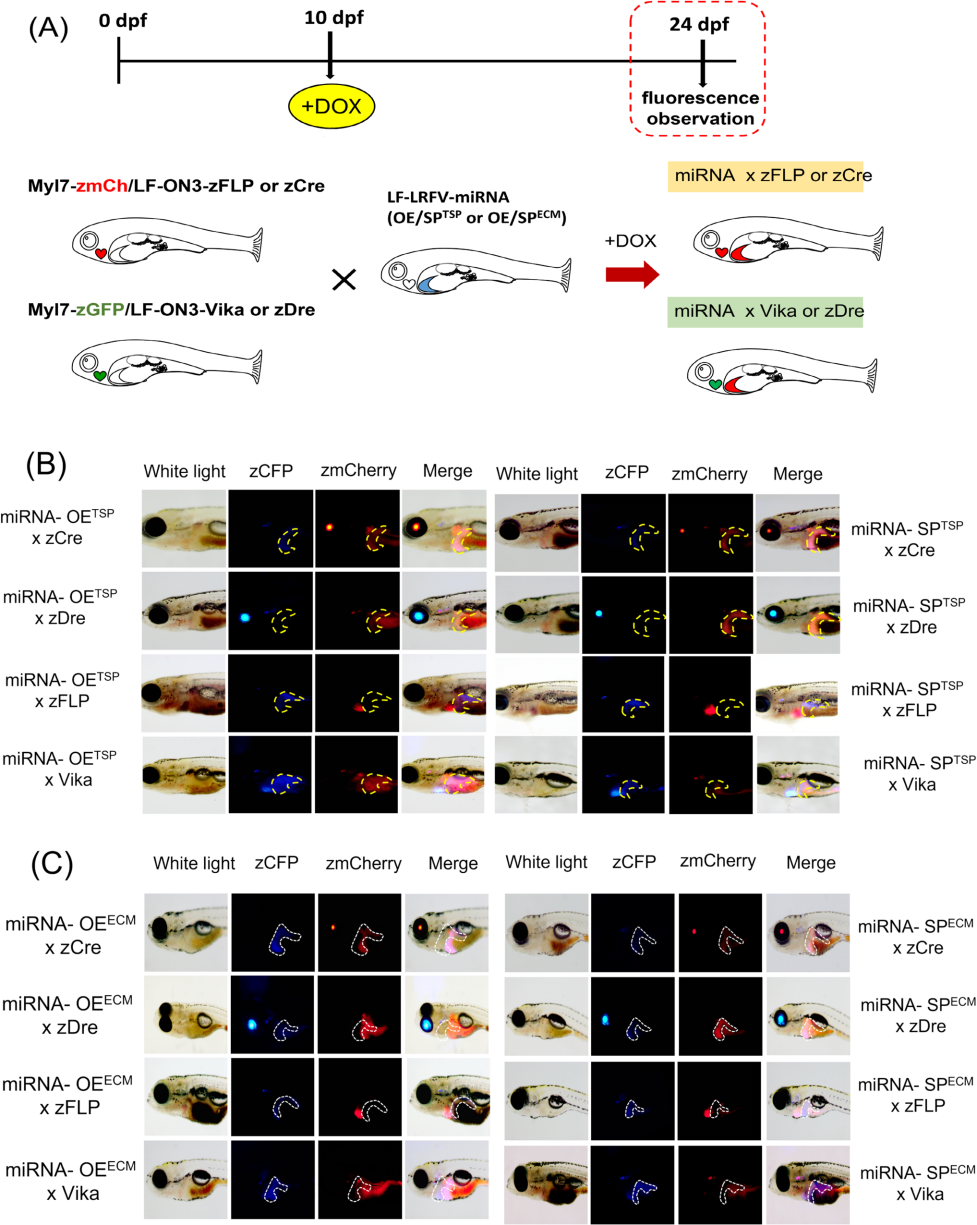
Induction of miRNA lines. **A** Experimental design of miRNA x zCre/zDre/zFLP/Vika expression line. Embryos were treated with 40 µg/ml DOX at 10 dpf, and the fluorescence was observed at 24 dpf. **B**, **C** The left image shows zebrafish larvae under white light, the middle image shows zCFP/zmCherry fluorescence, and the right image showed the overlap of white light and fluorescence. Red fluorescence signals were observed in the liver region in zCre and zDre groups after cross-breeding. Otherwise, zFLP and Vika groups show only a few red fluorescence signals. OE, overexpression; SP, sponge; TSP, TGF-β/SMAD pathway; ECM, extracellular matrix-associated pathway

**Fig. 4:**
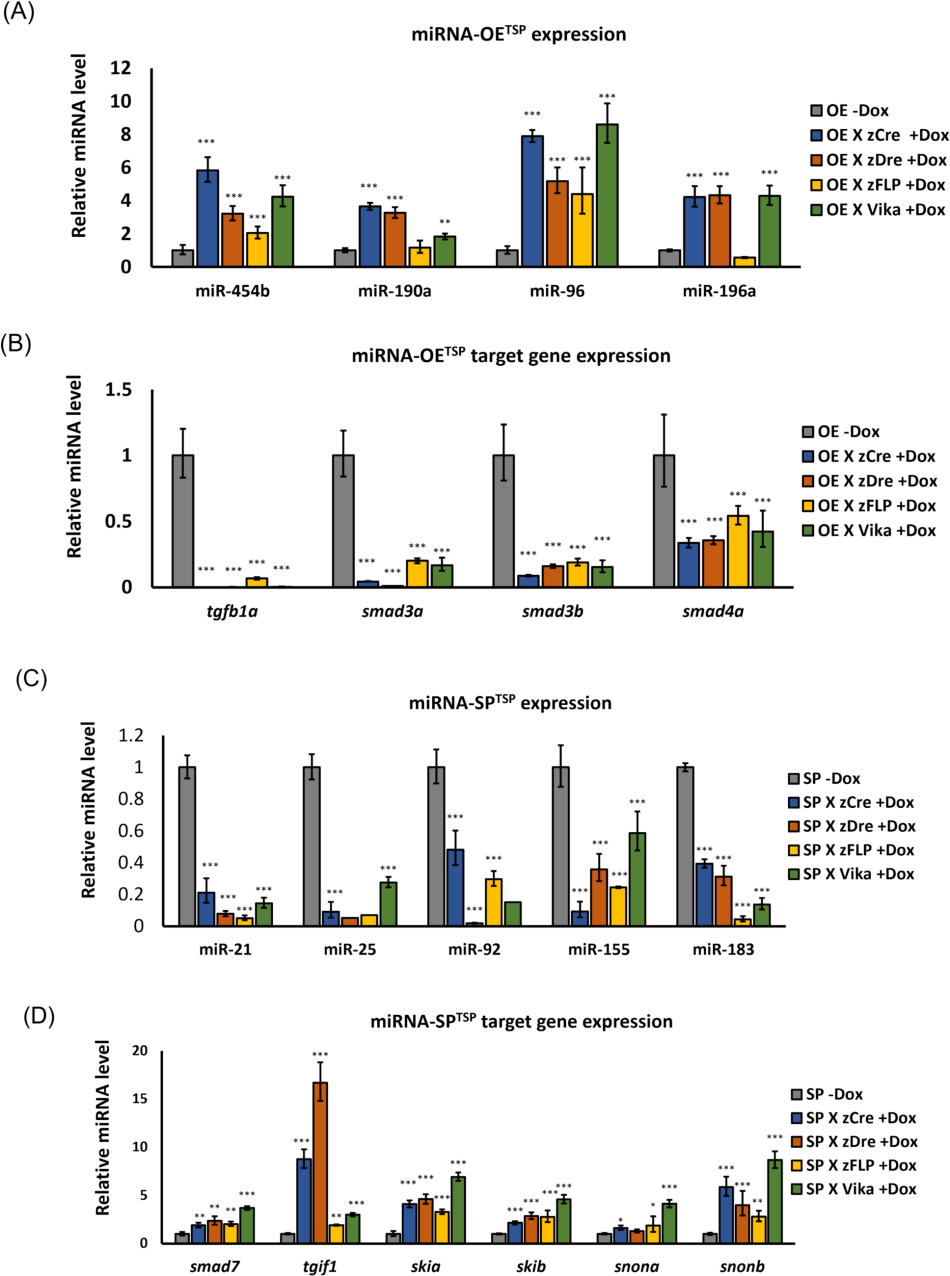
Expression levels of miRNA and target genes induced by recombinase. Larvae which were treated with doxycycline for 14 days and sacrificed at 24 dpf. Compare with the levels of miRNAs of miRNA-OE^TSP^ w/wo treating DOX. The expression level of **A** miRNA-OE^TSP^, **B** miRNA-OE^TSP^-targeted genes, **C** miRNA-SP^TSP^, and **D** miRNA-SP^TSP^-targeted genes (*n* = 3; *p < 0.05, **p < 0.01, ***p < 0.001). OE, overexpression; SP, sponge; TSP, TGF-β/SMAD pathway; ECM, extracellular matrix-associated pathway

**Fig. 5:**
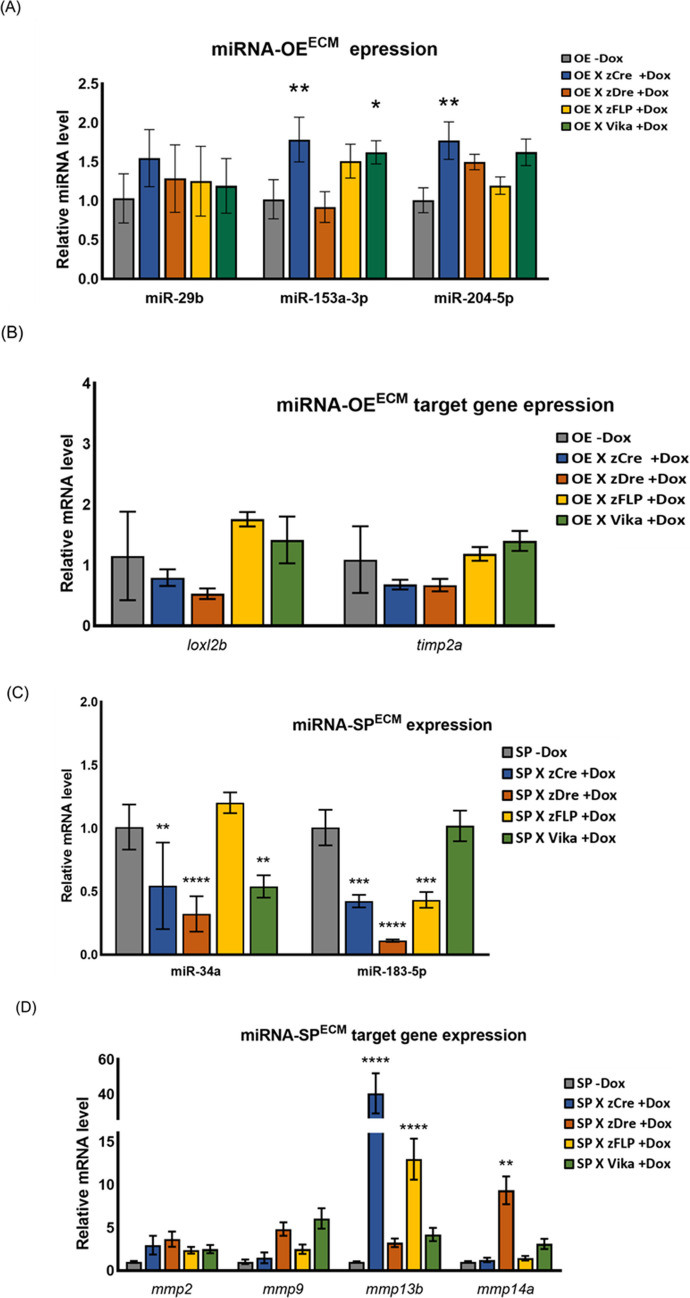
Expression levels of miRNA and target genes induced by recombinase. Larvae which were treated with doxycycline for 14 days and sacrificed at 24 dpf. Compare with the levels of miRNAs of miRNA-OE^ECM^ w/wo treating DOX. The expression level of **A** miRNA-OE^ECM^, **B** miRNA-OE^ECM^-targeted genes, **C** miRNA-SP^ECM^, **D** miRNA-SP.^ECM^-targeted genes (*n* = 3; *p < 0.05, **p < 0.01, ***p < 0.0001)

To establish tissue-specific zebrafish lines, constructs expressing miRNAs, eye (Cryb), and heart (Myl7)-specific promoters, liver (LF)-specific promoter following zGFP/zCFP/zmCherry fluorescence, and recognition sites for four recombinases (zCre/zDre/zFLP/Vika) with the Tet-ON3 inducible system were generated (Fig. 1A, S2). Each recombinase was labeled with a different fluorescent marker as follows: zCre was paired with red fluorescent protein (zmCherry), and zDre with green fluorescent protein (GFP) in the eyes. zFLP was paired with red fluorescent protein expressed, and Vika with green fluorescent protein in the heart. The constructs were microinjected into the cytoplasm of single-cell stage fertilized zebrafish eggs (Fig. 1B–E). At 5 dpf, the zebrafish (F0) were observed to produce fluorescence. Subsequently, the F0 zebrafish were allowed to continue rearing to generate the F1 generation. The producibility of fluorescence was observed in the F1 offspring, enabling the selection of stable transgenic lines, which express miRNAs with blue fluorescence in the liver (Fig. 2). The Tet-ON3 system was employed to induce the expression of recombinase. After crossing the four different types of recombinase zebrafish lines with the liver-specific miRNA-OE^TSP^, miRNA-SP^TSP^, miRNA-OE^ECM^, and miRNA-SP^ECM^ transgenic zebrafish lines, 40 µL/ml of DOX was added at 10 dpf to induce recombinase overexpression. We observed that the miRNA-expressing fish were successfully crossed with recombinase lines, which may express red fluorescence in the liver region (Fig. 3). The zCre and zDre recombinase lines showed more efficient activities compared to zFLP and Vika. Also, fish were sacrificed at 24 dpf for miRNA and targeted gene expression validation (Figs. 4 and 5). In the case of miRNA-OE^TSP/ECM^ lines, miRNAs were overexpressed after DOX induction (Figs. 4A and 5A), which led to their corresponding targeted gene being downregulated (Figs. 4B and 5B). On the other hand, while miRNA sponge functions as a loss-of-function tool, the miRNA-SP^TSP/ECM^ lines successfully induced the expression of the predicted miRNA, leading to the upregulation of its targeted genes (Figs. 4C, D and 5C, D).

### Optimization of miRNA expression in tgfβ1a-overexpressed fibrotic zebrafish model

**Fig. 6:**
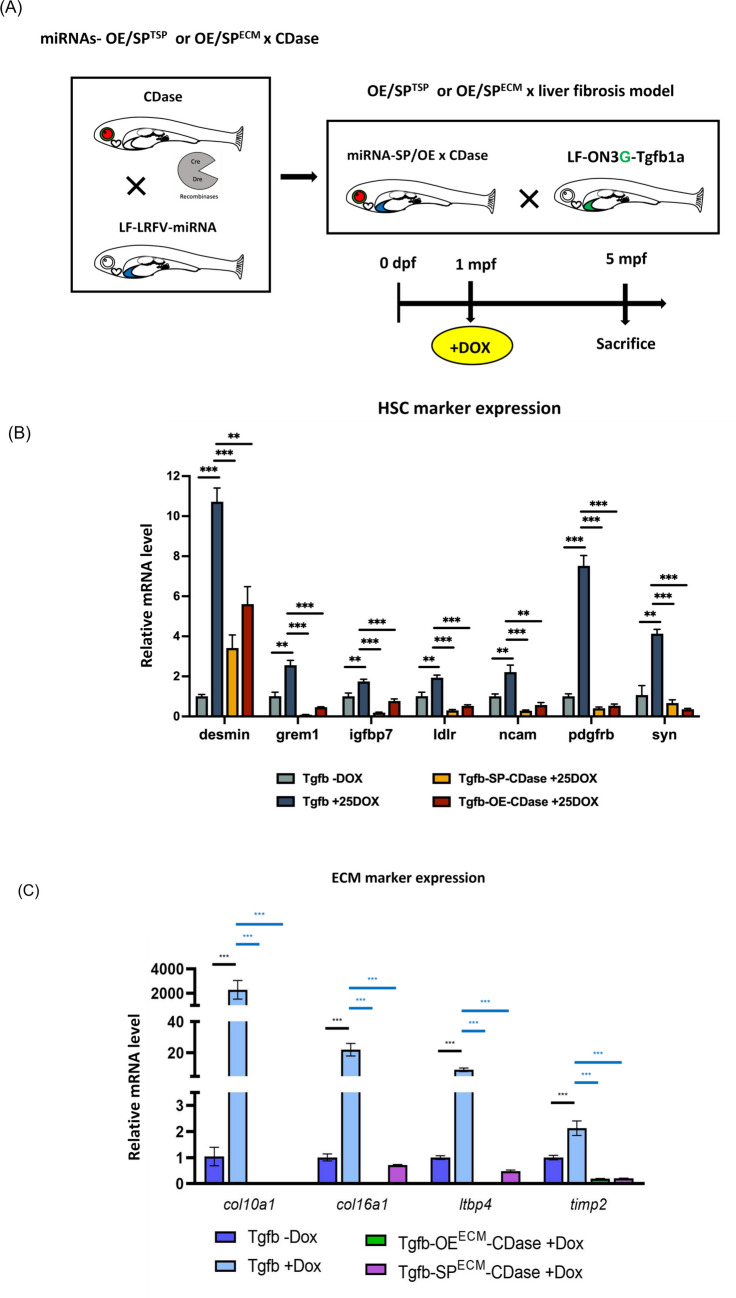

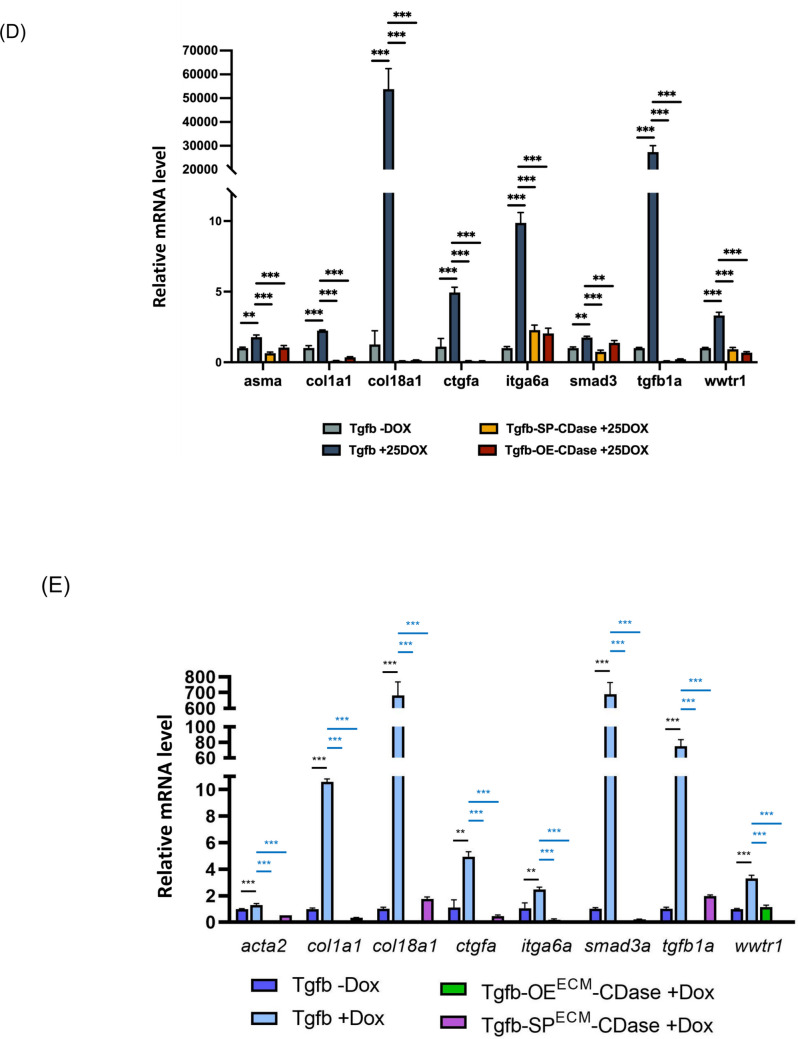
Generation and characterization of the liver fibrosis alleviation model through miRNA-mediated regulation of TGF-β1a signaling. **A** Scheme of breeding strategy of liver fibrosis alleviation model. Crossing of miRNA expression lines and double recombinase lines with an established TGF-β1a overexpressed line, which induced liver fibrosis. **B** Expression level of HSC markers. **C** Expression level of ECM markers. Expression levels of fibrosis markers in liver fibrosis alleviation model. To evaluate the impact of miRNA regulation on Tgf-β1a-induced fibrogenesis, we examined fibrosis biomarkers in the liver tissue. **D** In Tgfb-miRNA-OE/SP^TSP^ model. **E** In Tgfb-miRNA-OE/SP^ECM^ model (*n* = 3; **p < 0.01, ***p < 0.001). OE, overexpression; SP, sponge; TSP, TGF-β/SMAD pathway; ECM, extracellular matrix-associated pathway; CDase, dual-recombinase line, harbors zCre and zDre recombinase system

Following the successful validation of expression efficiency, a strategic genetic cross was employed to optimize the functional expression of microRNAs in a fibrotic zebrafish model induced by liver-specific overexpression of *tgfβ1a*. To achieve spatial and temporal control over miRNA activity, a dual-recombinase line, CDase, was established. This line harbors zCre and zDre recombinase systems, enabling refined inducible expression mechanisms through intersectional genetics. By integrating these recombinases in the offspring, precise regulation of miRNA expression in designated tissues was accomplished. Concurrently, to establish a pathophysiologically relevant fibrotic environment, a liver-specific tgfβ1a-inducible zebrafish model—previously regenerated in our laboratory—was employed [39]. This model permits controlled activation of the TGF-β signaling cascade, a known central pathway in hepatic fibrosis progression. The combination of CDase and the tgfβ1a model via genetic crossing enabled the generation of experimental cohorts with inducible liver fibrosis and targeted miRNA modulation, as schematized in Fig. 6A. This dual-recombinase system design is particularly advantageous for dissecting the temporal dynamics and tissue specificity of microRNA involvement in fibrogenic pathways, especially given the role of dysregulated miRNAs in the etiology of liver fibrosis as previously reported in mammalian systems [40, 41]. Thus, recent advances in CDase-mediated miRNA and sponge delivery support the feasibility of stable gene knockdown or overexpression through engineered miRNA backbones, which align with the modular flexibility the CDase platform offers. This integrated approach provides a robust in vivo system for elucidating the mechanistic roles of miRNAs in fibrotic signaling and evaluating therapeutic candidates targeting miRNA-regulated fibrogenesis.

### Selective miRNAs suppressed TGF-β-associated signaling

At 1 month-post-fertilization (mpf), the fibrotic lines were induced with 40 µg/mL DOX for another 4 months. Liver tissue was collected at 5 mpf for qPCR analysis to validate the selective miRNAs’ effects on *tgfβ1a*-associated fibrotic signaling. We first investigated the expression of seven HSC markers, including *desmin*, *grem1*, *igfbp7*, *ldlr*, *ncam*, *pdgfrb*, and *syn*. ECM-associated markers were also examined, including *col10a1a*, *col16a1*, *itbp4*, and *timp2* (Fig. 6B, C). Compared to the control group, the group with DOX induction exhibited significant upregulation of HSC markers and TGF-β/SMAD pathway-associated genes, which indicated that overexpression of TGF-β leads to HSC activation. However, when miRNAs exerted their inhibitory effects, the SP^TSP^ and OE^TSP^ groups exhibited a significant downregulation of HSC markers compared to the PC group. This suggests that our predicted miRNAs effectively suppressed TGF-β/SMAD signaling via the underlying genetic network. Furthermore, we also examined ECM-related biomarkers. We found that the expression levels of *col10a1a*, *col16a1*, *ltbp4*, and *timp2* were significantly lower in the SP^ECM^ and OE^ECM^ groups, which indicated that our selective miRNAs effectively inhibit fibrosis through ECM and TGF-β-associated ECM regulation (Fig. 6C). Finally, we examined myriad fibrosis markers to evaluate the miRNA-associated attenuation of liver fibrosis. TGF-β/SMAD pathway-associated genes, including *asma*, *col1a1*, *col18a1*, *ctgfa*, *itga6a*, *smad3*, and *wwtr1*, were also investigated (Fig. 6D, E). The widespread influence of miRNA-OE or SP through HSC and ECM-associated networks significantly decreased the incidence of liver fibrosis.

**Fig. 7:**
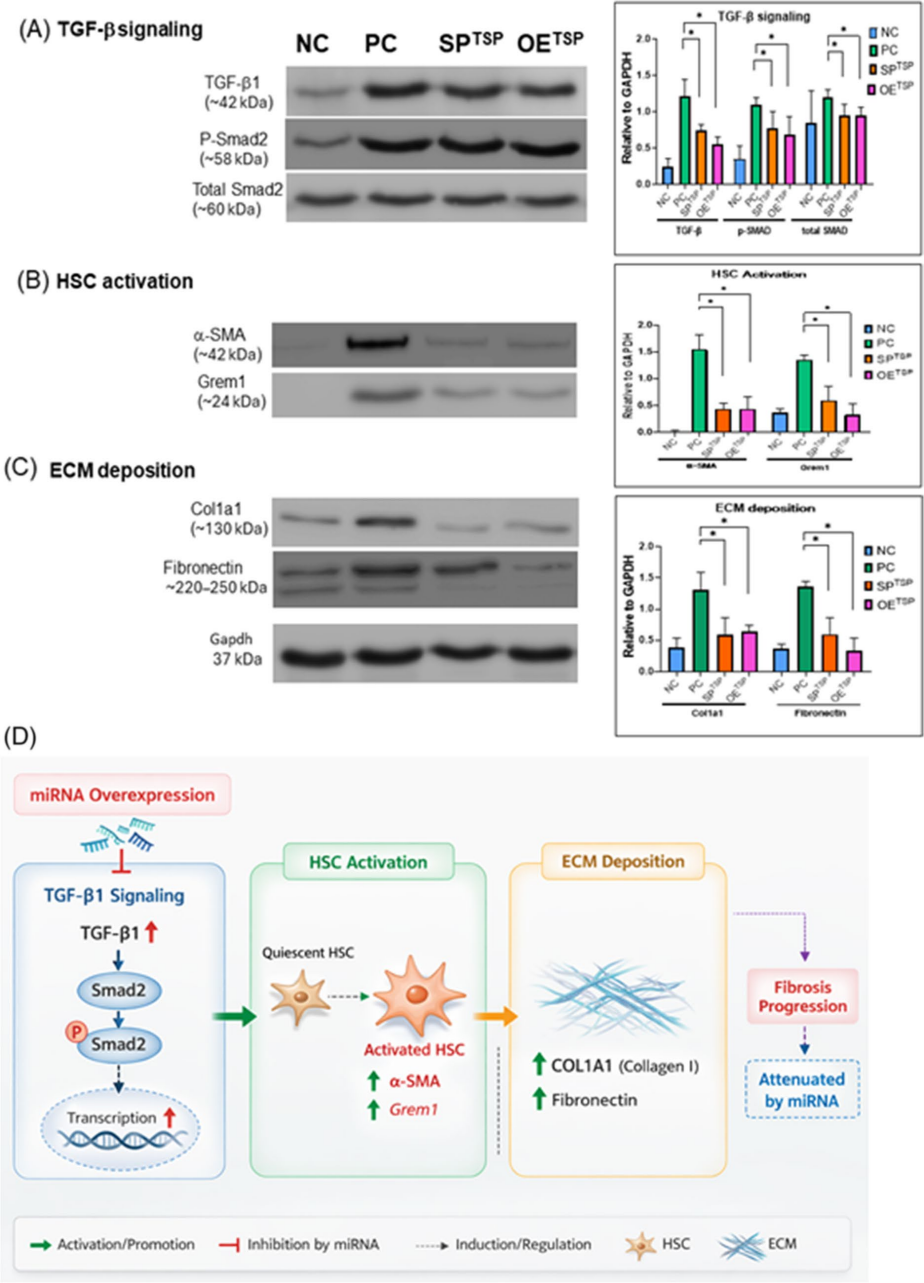
MicroRNAs attenuate fibrotic protein expression through inhibition of TGF-β1 signaling in Tgfb-OE/SPTSP zebrafish. Western blot analysis was performed to evaluate representative proteins associated with **A** TGF-β signaling, including TGF-β1, phosphorylated Smad2 (p-Smad2), and total Smad2; **B** hepatic stellate cell (HSC) activation markers, including α-smooth muscle actin (α-SMA) and Gremlin1 (Grem1); and **C** extracellular matrix (ECM) deposition markers, including collagen type I alpha 1 (COL1A1) and fibronectin. Representative immunoblots are shown in the left panels, and corresponding densitometric quantification is presented in the right panels. **D** Schematic model illustrating that microRNAs suppress TGF-β/SMAD signaling, thereby attenuating HSC activation and ECM accumulation during hepatic fibrogenesis. Adult zebrafish were treated with doxycycline (DOX; 40 µg/mL) for 4 months and sacrificed at 5 months post-fertilization for protein analysis. NC, Tgfb-OE without DOX; PC, Tgfb-OE with DOX; SP, Tgfb-SP-CDase+DOX; OE, Tgfb-OE-CDase+DOX. Protein expression was normalized to GAPDH. ECM, extracellular matrix; OE, overexpression; SP, sponge

Moreover, the protein level of TGF-β1 and GREM1 also demonstrated that the effectiveness of miRNA targeting inhibition may lead to reversing the HSC activation (Fig. 7A). Also, one of the major components in ECM, aka profibrotic marker, α-SMA, was reduced after SPECM or OEECM inhibition (Fig. 7B). Importantly, protein-level validation further confirmed that miRNA modulation suppresses phosphorylation of Smad2, a key downstream mediator of TGF-β signaling, accompanied by reduced expression of ECM structural proteins such as collagen I and fibronectin (Fig. 7A–C). These findings reinforce the notion that miRNAs exert antifibrotic effects by simultaneously targeting multiple nodes within the TGF-β/SMAD–HSC–ECM regulatory axis.

Taken together, these protein-level analyses provide mechanistic evidence that miRNA-mediated regulation suppresses hepatic fibrosis by inhibiting TGF-β/SMAD signaling, preventing hepatic stellate cell activation, and reducing ECM deposition (Fig. 7).

### The SP^TSP^, OE^TSP^ involved in the suppression of HSC activation and SP^ECM^, OE^ECM^ reduce ^ECM^ deposition to facilitate the regression of hepatic fibrosis in zebrafish

**Fig. 8:**
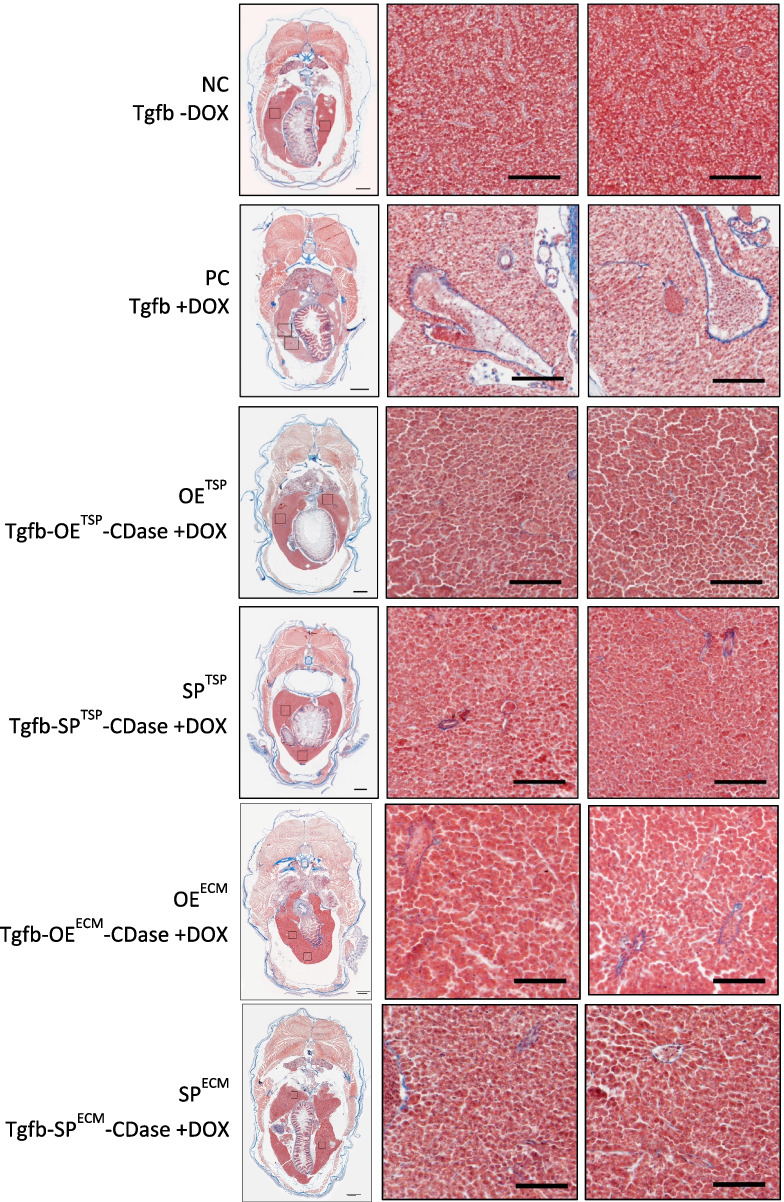

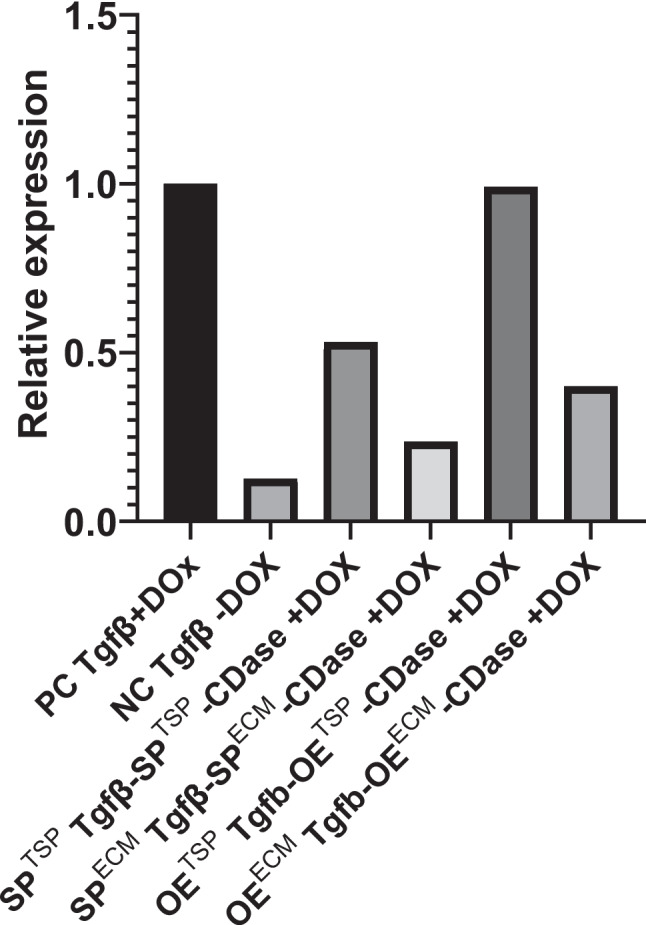
Masson’s trichrome staining of liver fibrosis alleviation model at 5 months post-fertilization (mpf). Zebrafish at 1 mpf were treated with doxycycline (DOX; 40 µg/mL) for 4 months and sacrificed for Masson’s trichrome staining. Blue staining indicates collagen deposition, reflecting the extent of liver fibrosis. Representative images are shown in the upper panels, and corresponding quantification of collagen-positive areas is presented in the lower panels (*n* = 3). Scale bars: 500 µm (whole liver images) and 100 µm (magnified images). OE, overexpression; SP, sponge; TSP, TGF-β/SMAD pathway; ECM, extracellular matrix; CDase, dual-recombinase line harboring zCre and zDre recombinase systems

**Fig. 9:**
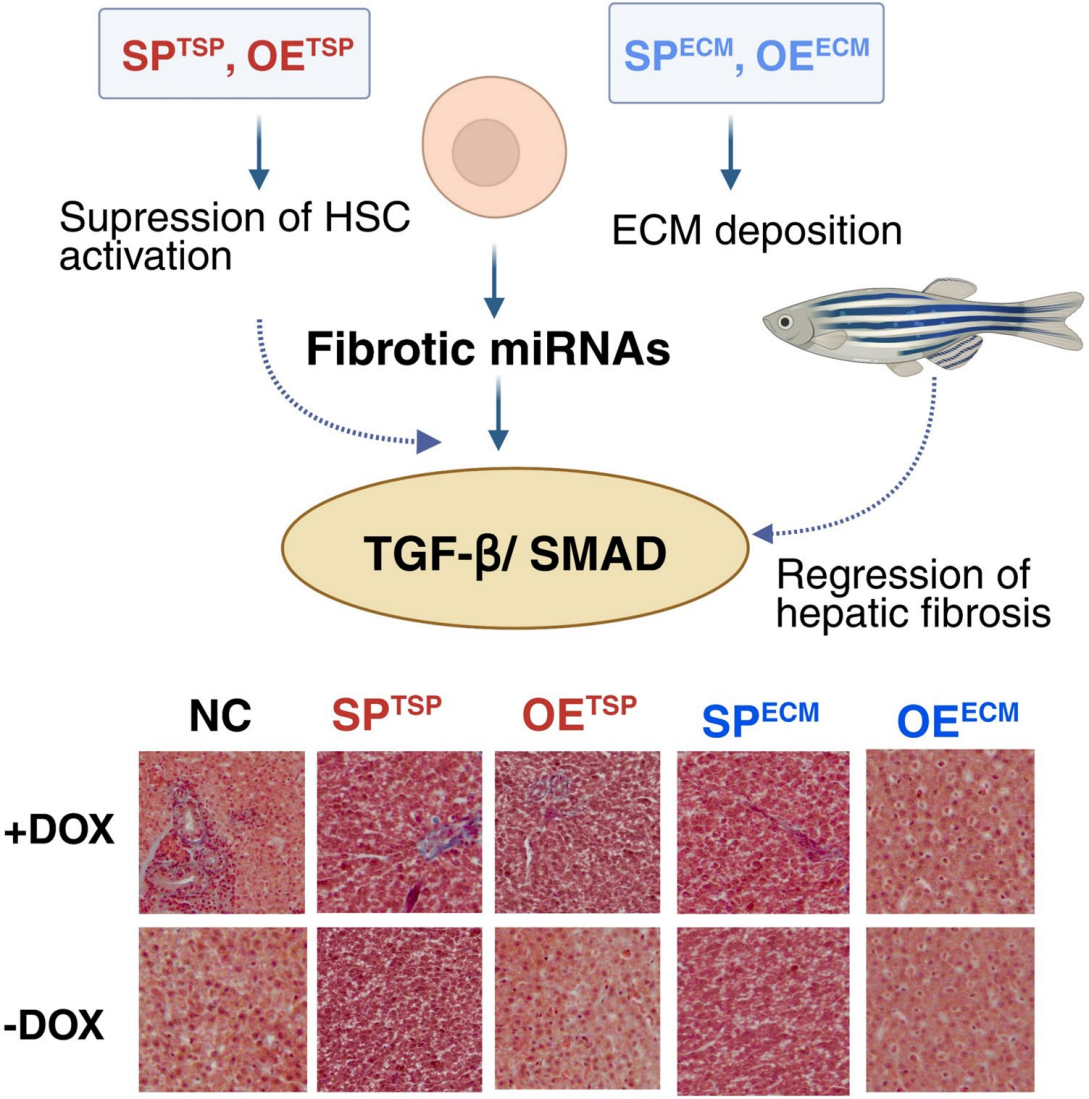
MiRNAs attenuate hepatic fibrosis via suppression of TGF-β/SMAD signaling in zebrafish. Masson’s trichrome staining reveals that SPTSP, OETSP, SPECM, and OEECM treatments reduce collagen deposition (blue staining) and restore hepatic architecture compared to the fibrotic model. These findings indicate that miRNAs suppress hepatic stellate cell (HSC) activation and extracellular matrix (ECM) accumulation, thereby promoting the regression of liver fibrosis. OE, overexpression; SP, sponge; TSP, TGF-β/SMAD pathway; ECM, extracellular matrix

Building on previous results, it has been shown that miRNAs can effectively inhibit hepatic HSC activation and ECM composition by modulating the TGF-β/SMAD signaling pathway through miRNA inhibition. To further explore this, we examined changes in liver tissue morphology and structure using histological staining, which highlights various cell types and collagen protein deposition. Fish were sacrificed at 5 mpf to observe histological changes. Masson’s trichrome staining demonstrated that the TGF-β-induced group (PC) exhibits a greater amount of blue-stained collagen fibers with less tightly packed cell arrangement, compared to the control group (NC). Disruption of hepatocyte arrangement with extended sinusoidal dilatation, and infiltration of immune cells with blue fibrotic signals spreading in tissues were observed. Conversely, the experimental groups (SP^TSP^, OE^TSP^, SP^ECM^, and OE^ECM^) exhibited fewer blue-stained collagen fiber signals, similar to the NC group, indicating reduced collagen fiber deposition and improved cell arrangement (Fig. 8). These findings directly indicated that miRNAs-mediated regulation can suppress HSC activation and reduce ECM deposition to facilitate the regression of hepatic fibrogenesis, thereby attenuating liver fibrosis and hepatic damage, which suggests their potential as therapeutic targets for liver cirrhosis (Fig. 9).

## Discussion

Liver fibrosis is a progressive pathological process characterized by sustained hepatic stellate cell (HSC) activation and excessive extracellular matrix (ECM) deposition, ultimately leading to architectural distortion and organ dysfunction. Among the signaling cascades implicated in fibrogenesis, the TGF-β1/SMAD pathway represents a central regulatory axis that coordinates HSC transdifferentiation, collagen synthesis, and ECM remodeling. In the present study, we established inducible, liver-specific microRNA (miRNA) transgenic zebrafish lines to investigate whether coordinated modulation of fibrogenic signaling networks could attenuate TGF-β1a–induced hepatic fibrosis. Our findings demonstrate that selective miRNA overexpression or sponge-mediated inhibition effectively suppresses HSC activation markers, reduces ECM-associated gene expression, decreases profibrotic protein levels, and improves histological architecture, collectively facilitating regression of hepatic fibrosis.

The zebrafish model offers distinct advantages for studying liver disease, including high genetic conservation with humans. Over 70% of human genes and approximately 80% of disease-associated genes have zebrafish orthologs, and many miRNAs are evolutionarily conserved across vertebrates. This high level of genetic counterpart makes zebrafish a valuable model for human pathology research. Moreover, most zebrafish miRNAs are highly conserved and functionally similar to those in humans, indicating their natural presence and activity across both species [42, 43]. Unlike siRNA-mediated single-gene silencing, miRNAs exert coordinated regulation across multiple targets within interconnected signaling networks. Hepatic fibrosis is a multifactorial process involving complex crosstalk between the TGF-β/SMAD axis, ECM remodeling pathways, inflammatory mediators, and paracrine signaling between hepatocytes and HSCs. Targeting a single molecule may therefore be insufficient to reverse fibrogenesis. In contrast, miRNAs provide a network-level regulatory mechanism capable of simultaneously modulating several fibrogenic mediators. Furthermore, dysregulation of specific miRNAs has been widely reported in human liver fibrosis, underscoring their physiological relevance and therapeutic potential. Thus, our strategy aimed to harness endogenous miRNA regulatory properties to achieve broader suppression of fibrogenic signaling rather than isolated gene inhibition. However, while this genetic model allows precise temporal control of fibrosis induction, it may not fully recapitulate the complex etiologies of human liver fibrosis, such as alcohol-associated liver disease or toxin-induced injury. Future studies utilizing ethanol immersion or thioacetamide (TAA)-induced fibrosis models in zebrafish will be important to validate the antifibrotic efficacy of the identified miRNAs under more physiologically relevant conditions.

To enable spatial and temporal precision, we developed a liver-specific inducible platform using the fabp10a (L-FABP) promoter combined with Tet-ON3 and dual-recombinase systems. This design allowed controlled miRNA expression in hepatocytes following doxycycline induction. Although HSCs are the principal effector cells driving fibrosis, hepatocytes serve as a major source of profibrotic mediators, including TGF-β1, which act in a paracrine manner to activate HSCs. Therefore, hepatocyte-specific miRNA modulation may indirectly attenuate HSC activation by reshaping the fibrogenic microenvironment. The observed reduction in HSC markers (*desmin*, *pdgfrb*, *grem1*, *igfbp7*, *ncam*, *syn*) and α-SMA protein levels supports this indirect regulatory mechanism. Nevertheless, we acknowledge that HSC-specific promoter systems could provide additional mechanistic precision. Future investigations incorporating HSC-targeted expression strategies will help delineate cell-autonomous versus paracrine miRNA effects in fibrogenesis.

Our results further demonstrate that selective miRNAs targeting ECM-associated pathways significantly reduced collagen deposition and expression of key ECM remodeling genes, including *col10a1a*, *col16a1*, *ltbp4*, *and timp2*. The interplay between MMPs and TIMPs is critical in maintaining ECM homeostasis, and dysregulation of this balance contributes to persistent fibrosis. By modulating multiple nodes within the ECM regulatory network, miRNA intervention appears to restore matrix equilibrium. Histological analyses via Masson’s trichrome staining revealed markedly decreased collagen accumulation in miRNA-regulated groups compared with TGF-β1a-induced controls, confirming functional attenuation of fibrotic remodeling.

Additionally, the Tol2 transposon, which is endogenous to zebrafish, can be utilized to create an efficient gene transfer system, facilitating the regulation of gene expression [44]. Moreover, due to zebrafish generating transient fibrosis with canonical biological pathways, it is known to be an ideal model for antifibrotic therapeutic application for the next clinical stage [45]. Our results indicate that zFLP and Vika recombinases exhibit significantly lower recombination efficiency compared to zCre and zDre. This disparity may stem from intrinsic differences in the recombinase enzymes or variations in the efficiency of plasmid integration into the zebrafish genome. However, other contributing factors may not be ruled out. The Cre/loxP recombination system employs the tyrosine recombinase Cre and is extensively utilized in mammalian models due to its robust efficiency and precise site-specific recombination at 34-base pair (bp) loxP recognition sites in vivo [46]. In parallel, the Dre/rox system—derived from bacteriophage D6—exhibits structural and functional homology to Cre, with the rox recognition site sharing 21 nucleotides with loxP, which facilitates orthogonal recombination [47]. We cross-bred the zCre and zDre recombinases to optimize the expression, which maintained distinct specificities for their lox and rox target sites, respectively. The combined and sequential activation enables precise temporal control of transgene activation and inactivation to apply the following examinations. Notably, shorter induction periods revealed more pronounced differences in recombination efficiency, whereas longer induction times appeared to mitigate these differences (data not shown). Therefore, future experiments may be performed to extend the induction duration to 2–4 months for more consistent results if necessary.

Previously, studies showed that a number of genes that regulate ECM turnover are regulated through miRNAs. Transfection of miR-146a significantly suppresses MMP-13 in human chondrocytes, which is associated with cartilage regeneration [48]. On the other hand, miR-125a and miR-125b demonstrated suppressive activity on ERBB2/3 in human breast cancer cell lines, which were known for being pivotal cytokines and regulating ECM assembly [49]. Our results suggest that the SP^ECM^ clone reduces MMP-14 expression and may significantly inhibit the ECM pathway via miR-125b. Moreover, one of our predicted miRNA targets, miR-34a, was validated in prostate cancer metastasis as a direct regulator of CD44, which was a cell surface receptor involved in ECM components [50, 51]. This suggests that miR-34a may mediate cell–cell and cell-ECM interactions through multiple targets. One of the key members of the TIMP family, TIMP2, a canonical inhibitor of MMPs that can degrade the ECM structure, which were regulated by myriad miRNAs, such as miR-429 [52], miR-106a [53], miR-205-5p [54], miR-210 [55], indicated its significant role in ECM-dysregulated diseases. We designed miR-153a and miR-204 sequences within OE^ECM^, which demonstrated inhibitory effects on *timp2*, and recovery of liver fibrosis, which inferred the success of our prediction and the effectiveness of miRNAs. By incorporating our systematic transgenic model, miRNAs’ precise and spatial regulation will elucidate liver fibrosis mechanisms and support comprehensive therapeutic strategies in future clinical applications.

Although histological and molecular analyses demonstrated significant attenuation of fibrosis following miRNA modulation, biochemical indicators of liver injury and function, such as alanine aminotransferase (ALT) and aspartate aminotransferase (AST), were not assessed. Due to the limited blood volume obtainable from adult zebrafish, serum-based enzymatic assays remain technically challenging. Consequently, the current conclusions are based on molecular and histopathological endpoints. Future investigations employing larger vertebrate models will be necessary to determine whether miRNA-mediated antifibrotic effects translate into measurable improvements in liver function. Another important consideration is translational validation. While zebrafish provide a powerful in vivo platform for genetic and mechanistic studies, confirmation in mammalian systems is essential for clinical advancement. Future studies will evaluate the functional effects of selected miRNAs and miRNA sponges in human hepatic stellate cell lines, such as LX-2 cells, to determine whether conserved antifibrotic mechanisms operate across species. Such cross-species validation will strengthen the therapeutic potential of miRNA-based strategies targeting the TGF-β/SMAD axis and ECM remodeling pathways.

Importantly, protein-level validation further confirmed that miRNA modulation suppresses phosphorylation of Smad2, a key downstream mediator of TGF-β signaling, accompanied by reduced expression of extracellular matrix proteins, including collagen I and fibronectin. These coordinated effects across signaling, cellular activation, and matrix deposition strongly support the concept that miRNAs exert antifibrotic activity through simultaneous targeting of multiple nodes within the TGF-β/SMAD–HSC–ECM regulatory axis.

In summary, this study establishes a robust inducible zebrafish platform for spatially controlled miRNA modulation and demonstrates that coordinated regulation of TGF-β/SMAD signaling and ECM-associated pathways effectively attenuates hepatic fibrosis. By leveraging the intrinsic network-regulatory properties of miRNAs, our approach provides a promising framework for antifibrotic therapeutic development. Continued validation in physiologically relevant injury models and mammalian systems will further define the translational potential of miRNA-based interventions in chronic liver disease.

## Supplementary Information

Below is the link to the electronic supplementary material.

ESM1 (DOCX 632 KB)

## Data Availability

The authors confirm that the data supporting the findings of this study are available within the article.
