# Supplementary material for: Fibrotic microRNAs in the suppression of HSC activation and ECM deposition to facilitate the regression of hepatic fibrosis in zebrafish
Source: J Mol Med (Berl). 2026 Apr 30;104(1):70. doi: 10.1007/s00109-026-02672-y (PMC13133201; doi:10.1007/s00109-026-02672-y)
Supplement: Supplementary file 1 — (DOCX 632 KB) [file 109_2026_2672_MOESM1_ESM.docx]

Table S1. Sequences of qRT-PCR primers.

| **Housekeeping gene** | | |
| --- | --- | --- |
| ***gapdh*** | Forward | GTGGAGTCTACTGGTGTCTTC |
|  | Reverse | GTGCAGGAGGCATTGCTTACA |
| ***U6*** | Forward | TTGGTCTGATCTGGCACATATAC |
|  | Reverse | AAAAATATGGAGCGCTTCACG |
| **miRNAs** | | |
| **miR-21** | Forward | GTTTGTAGCTTATCAGACTGGTG |
| **miR-25** | Forward | CATTGCACT TGTCTCGGTCTGA |
| **miR-92a** | Forward | TATTGCACTTGTCCCGGCCTGT |
| **miR-96** | Forward | TTTGGCACTAGCACATTTTTGCT |
| **miR-190a** | Forward | GATATATTAGGTGTTGGCTCTG |
| **miR-196a** | Forward | TAGGTAGTTTCATGTTGTTGGG |
| **miR-155** | Forward | TTAATGCTAATCGTGATAGGGGT |
| **miR-183** | Forward | TATGGCACTGGTAGAATTCACT |
| **miR-454b** | Forward | TAGTGCAATATTGCTTATAGGG |
| **miR-29b** | Forward | TAGCACCATTTGAAATCAGTGT |
| **miR-34a** | Forward | TGGCAGTGTCTTAGCTGGTTGT |
| **miR-125b-5p** | Forward | AACTTGTGAGTTGGCTCTGGTG |
| **miR-150** | Forward | TCTCCCAATCCTTGTACCAGTG |
| **miR-153a-3p** | Forward | TTGCATAGTCACAAAAGTGATC |
| **miR-183-5p** | Forward | TATGGCACTGGTAGAATTCACTG |
| **miR-193a-3p** | Forward | GCCTACAAAGTCCCAGTGTTG |
| **miR-204-5p** | Forward | TTCCCTTTGTCATCCTATGCCT |
| **miRNAs target gene** | | |
| ***tgfβ1a*** | Forward | CAATCCTTGACCCTCTGCC |
|  | Reverse | TCGAGCCATGAACCACTTTC |
| ***smad3a*** | Forward | GGAGAAATGGTGCGAAAAGG |
|  | Reverse | CTTGCAATCGACCGTCTAGAG |
| ***smad3b*** | Forward | TCTGCTCTCCAATGTCAACC |
|  | Reverse | ACTCTGGACGAAAATAGCACTG |
| ***smad4a*** | Forward | AGCAACGTCCACAGAACAG |
|  | Reverse | CTCCTTCACCTTTACACTCCAG |
| ***smad7*** | Forward | ACCAGACAGTTCATCAGCAG |
|  | Reverse | ATCCTCTGGAGAACAAACGG |
| ***tgif1*** | Forward | GGGAATTTTGACGGACATGAAG |
|  | Reverse | TCCTCTTCCTCTTCCCTGA |
| ***skia*** | Forward | CAGGAGACAAAGACAAGCAAC |
|  | Reverse | TGCTCCGTCTTTATCGCTAAC |
| ***skib*** | Forward | GCATACAGTCCTATTCCCTCC |
|  | Reverse | AAGGTCTCTGCTTGAACTGG |
| ***snona*** | Forward | CATCAAAGAGGAACCAGGACA |
|  | Reverse | GAACGTCCTCCTGCTGTAGTG |
| ***snonb*** | Forward | AAGTTCCAGCAGGAGAAGAGG |
|  | Reverse | GTGTTGTGCCAAACCATTACC |
| ***loxl2b*** | Forward | GATTTCACCATTCATGCAGCC |
|  | Reverse | CTTCTCCTTTCCCTTACAGTGG |
| ***mmp2*** | Forward | ATCATAAAGTTCCCCGGCG |
|  | Reverse | TCTTCAGCGTGTCCTTCAAC |
| ***mmp9*** | Forward | ACAGAAGGACGAAATGACGG |
|  | Reverse | GGAAGGTGAATGGAAAATGGC |
| ***mmp13b*** | Forward | ACGAGGATGAAACATGGACTG |
|  | Reverse | TTTGGCGTAGGAGTAAACCG |
| ***mmp14a*** | Forward | ACCAGATAATGTCAAAGGAGCC |
|  | Reverse | CAGCCCATCCAGTTAGTGAG |
| ***timp2a*** | Forward | CAGCCCATCCAGTTAGTGAG |
|  | Reverse | CTTCTCCGTCACCCAATCTG |
| **HSC marker** | | |
| ***desmin*** | Forward | AATGAAAGACGAGATGGCCC |
|  | Reverse | AGGGATGAAAAGGACTGCAC |
| ***grem1*** | Forward | TCATCCACGCTGACAGTTATG |
|  | Reverse | TTTGTCTGGATGCGGGATG |
| ***igfbp7*** | Forward | AAGATAATCTGGCGGTGCAG |
|  | Reverse | GATCCGTCCTCAGCTTTAGTG |
| ***ldlr*** | Forward | CAAGATCCCTAACAGACCCAC |
|  | Reverse | AGAGACAGCCTTAACATCACC |
| ***n-cam*** | Forward | TCATCGGCACAGAGTCAAAG |
|  | Reverse | GTACTTGAGAACAGGCACTCC |
| ***pdgfrb*** | Forward | CAGTGATTCTCCGGTTCTCAG |
|  | Reverse | CAGTTTGCCTTCACAGATCAG |
| ***syp*** | Forward | TTTACGACCCTGTCATGTCTG |
|  | Reverse | GTTACCCGTCCACAAGATCAG |
| **ECM marker** | | |
| ***col10a1a*** | Forward | AGTATGCCGATTTGACCTCAG |
|  | Reverse | TTTTCACGGAGTAAGGCTGG |
| ***col16a1*** | Forward | TGTATTGTGACCCCTCTCTGG |
|  | Reverse | ACATTTTTCCGCCAGTTCTTT |
| ***ltbp4*** | Forward | TCATTCAAGCCCAGACCATAC |
|  | Reverse | GAGCGTACACTCGTTGATTCC |
| ***timp2a*** | Forward | AATCCATGAGTGCCACCC |
|  | Reverse | CTTCTCCGTCACCCAATCTG |
| **Fibrosis marker** | | |
| ***asma*** | Forward | GCTCTTCCACATGCCATTATG |
|  | Reverse | ACGTAGCACAACTTCTCCTTG |
| ***acta2*** | Forward | GCTCTTCCACATGCCATTATG |
|  | Reverse | ACGTAGCACAACTTCTCCTTG |
| ***col1a1*** | Forward | CAAGGGACACAGAGGATTCAG |
|  | Reverse | ATCATTACCACGAGCACCAG |
| ***col18a1a*** | Forward | GAGGATGACTATGCAACAGAGG |
|  | Reverse | TTGGCTGAACTGGTGATGG |
| ***ctfgα*** | Forward | CTTTACTGTGACTACGGCTCC |
|  | Reverse | ACAACTGCTCTGGAAAGACTC |
| ***itga6a*** | Forward | TCACACCTGAGCACATTCTG |
|  | Reverse | CACCAACTTCCTCATCCCTC |
| ***smad3a*** | Forward | GGAGAAATGGTGCGAAAAGG |
|  | Reverse | CTTGCAATCGACCGTCTAGAG |
| ***tgfβ1a*** | Forward | CAATCCTTGACCCTCTGCC |
|  | Reverse | TCGAGCCATGAACCACTTTC |
| ***wwtr1*** | Forward | GACCCCTTCCTCAACAGTG |
|  | Reverse | TTTTCCCCTGTGTCCATGTC |


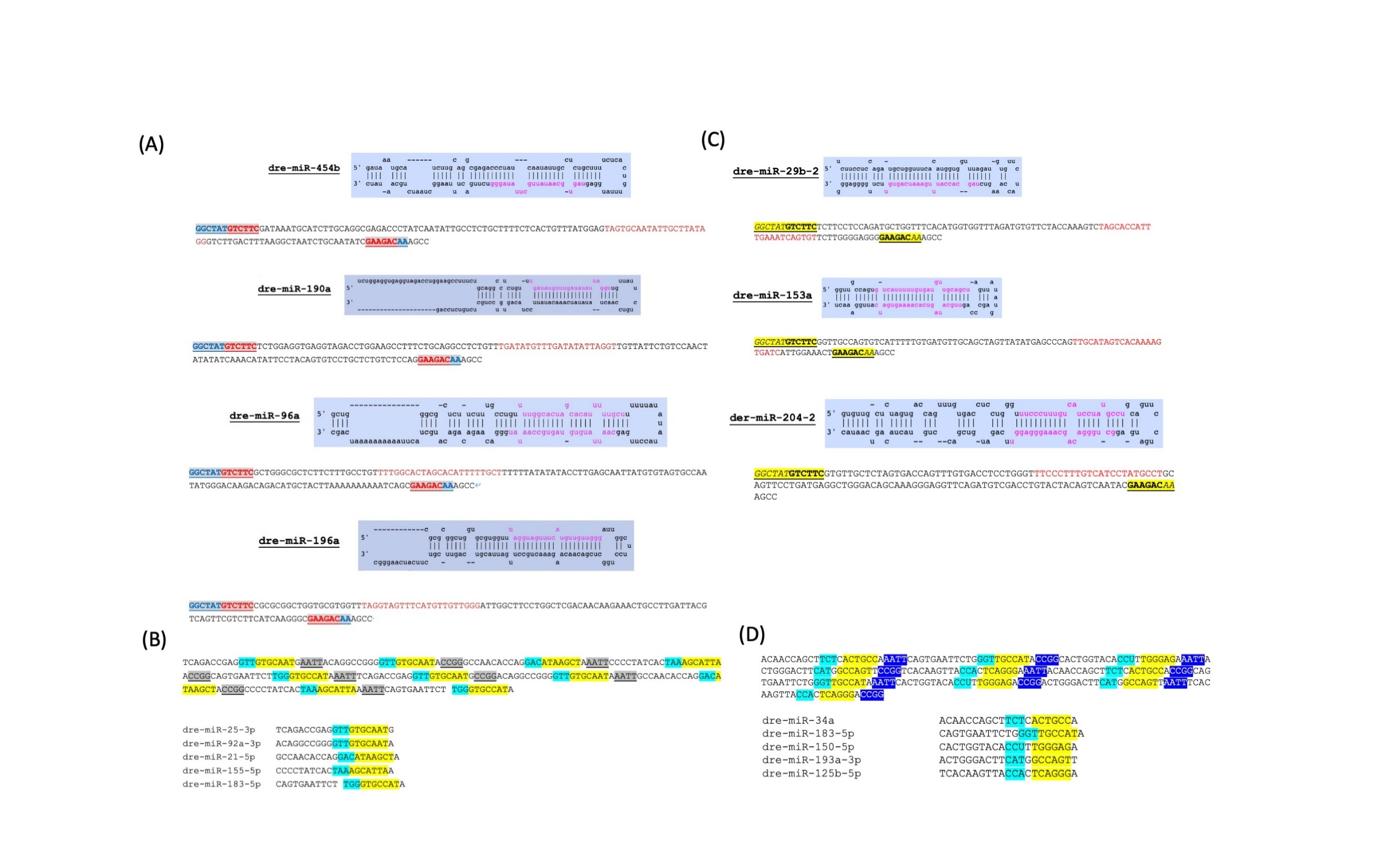


Fig. S1. MiRNA sequences of construct (A) LF-LRFV-miRNA-OE^TSP^ (B) LF-LRFV-miRNA-SP^TSP^ (C) ) LF-LRFV-miRNA-OE^ECM^ (D) LF-LRFV-miRNA-SP^ECM^.


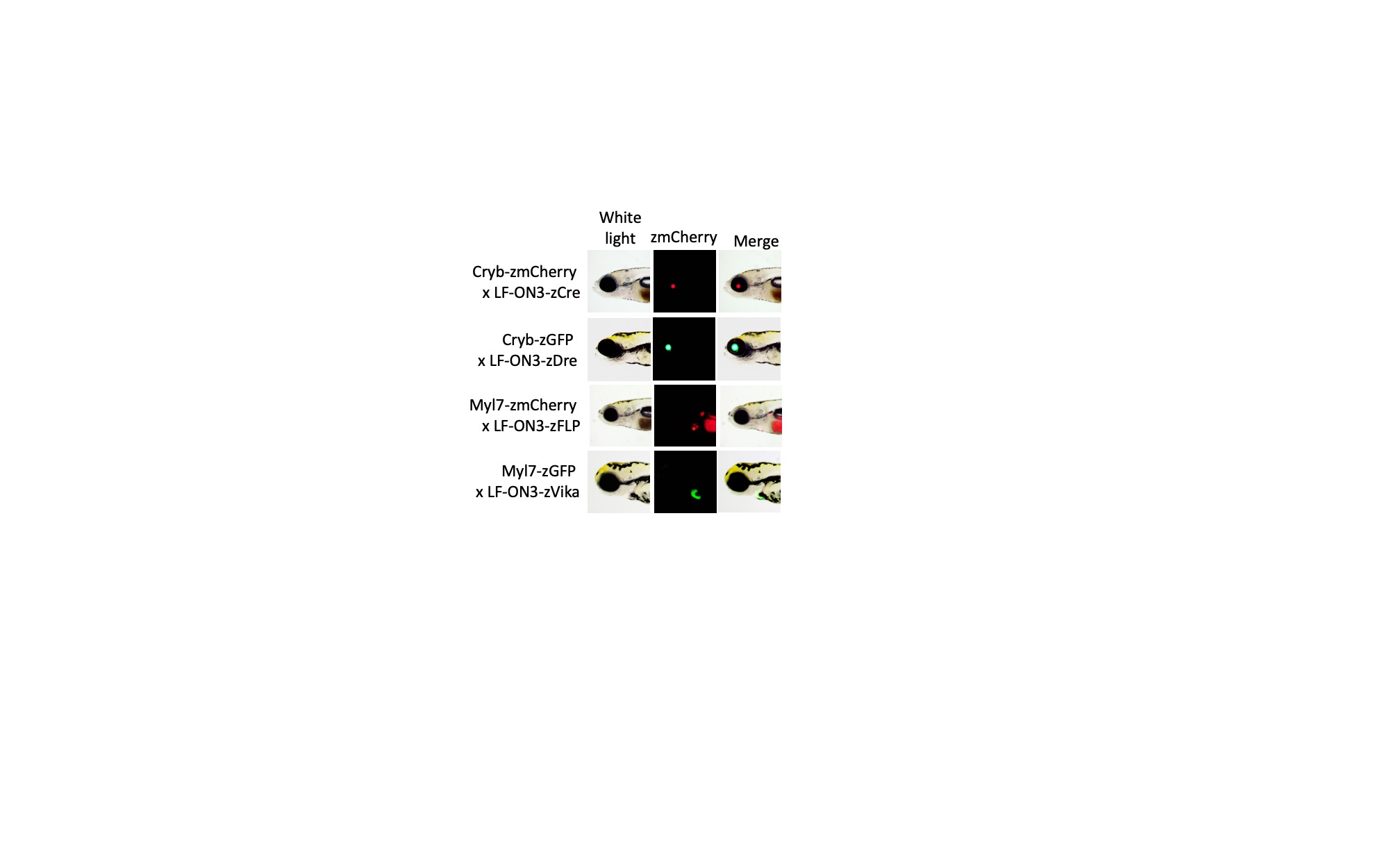


Fig. S2 Transgenic lines expressing the recombinases exhibited red and green fluorescence in the eyes and heart at 5-7 dpf. The left image shows zebrafish larvae under white light, the middle image shows zmCherry or zGFP fluorescence, and the right image shows the overlap of white light and fluorescence.
